# Mechanical and Plasma Electrolytic Polishing of Dental Alloys

**DOI:** 10.3390/ma16186222

**Published:** 2023-09-15

**Authors:** Katharina Witzke, Renko Kensbock, Caroline Ulrike Willsch, Katja Fricke, Sander Bekeschus, Hans-Robert Metelmann

**Affiliations:** 1 Department of Oral, Maxillofacial, and Plastic Surgery, Greifswald University Medical Center, Sauerbruchstr., 17475 Greifswald, Germany; renko.kensbock@med.uni-greifswald.de (R.K.); caroline.willsch@aol.de (C.U.W.); metelman@uni-greifswald.de (H.-R.M.); 2 Leibniz Institute for Plasma Science and Technology (INP), Felix Hausdorff-Str. 2, 17489 Greifswald, Germany; fricke@nebula-biocides.de (K.F.); sander.bekeschus@gmail.com (S.B.); 3 Clinic and Policlinic for Dermatology and Venerology, Rostock University Medical Center, Strempelstr. 13, 18057 Rostock, Germany

**Keywords:** dental alloy, surface roughness, conventional mechanical polishing, plasma electrolytic polishing, AFM, SEM, EDX

## Abstract

(1) Background: In dentistry, a reduction in surface roughness is established mostly by conventional mechanical polishing to hinder biofilm adhesion. This is time- and labor-intensive. Plasma electrolytic polishing is believed to be an effective finishing method due to the reduced treatment time and materials used for applications in dentistry. (2) Methods: Co-Cr-Mo dental alloy samples were sandblasted and prepared with either plasma electrolytic or conventional mechanical polishing. Evaluation of the polishing methods was obtained by atomic force microscopy, scanning electron microscopy and energy-dispersive X-ray spectroscopy. (3) Results: The sandblasted samples showed the highest surface roughness (Heraenium^®^ Sun 991 ± 288 nm; Wironit^®^ 1187 ± 331 nm). Our results show that with plasma electrolytic polishing, Co-Cr-Mo surfaces can be polished with a surface roughness in the nanometer range, comparable to those achieved by conventional mechanical polishing. Conventional mechanical polishing (Heraenium^®^ Sun 134 ± 23 nm; Wironit^®^ 114 ± 11 nm) provided lower surface roughness values compared to plasma electrolytic polishing (Heraenium^®^ Sun 288 ± 94 nm; Wironit^®^ 261 ± 49 nm). We anticipate our pilot study as a starting point for future studies to refine process parameters and quantitative microbiological assays. (4) Conclusions: Plasma electrolytic polishing might have a promising future for polishing dental alloys.

## 1. Introduction

Non-noble dental alloys are ubiquitous in dentistry and have gained usage in fixed and removable prosthodontics worldwide. These dental alloys consist of a Co-Cr-Mo basis. Co-Cr alloys have been available since 1929 [1]. A detailed knowledge of their structure is provided by Uriciuc et al. (2015) [2]. Biofilm formation, corrosion and surface roughness are connected and determine the lifespan of dental alloys in dentistry. The surface roughness of dental alloys is an important measure of biofilm adhesion and formation [3]. The oral biofilm is an intricately organized network of microorganisms and an extracellular matrix that strongly adhere to surfaces in the oral cavity [4]. This network ensures safety from harmful external influences such as host defenses or antibiotics, enabling deleterious bacterial growth under certain conditions [5,6]. Furthermore, secondary caries is a common reason for the replacement of restorations [7,8]. Hence, the metallic surface of dental alloys must be as smooth as possible to keep bacteria from adhering and hence limit biofilm formation [9]. A threshold surface roughness of about 0.2 µm has been proposed for optimum biofilm containment [10]. A typical bacterium in the oral cavity is Streptococcus mutans, which has a mean radius of 0.3 µm and is of comparable length [11].

Conventional mechanical polishing by a dental technician is the current standard method of preparing dental alloys [12]. In the literature, various efforts have been made to investigate the surface morphology and surface roughness of mechanically polished dental alloys [2,13,14]. Plasma electrolytic polishing is a novel and eco-friendly method of polishing alloys with reduced treatment time and required equipment [15,16]. The plasma electrolytic polishing system is equipped with a bath filled with an aqueous electrolyte like ammonium sulfate and a cathode. In general, a workpiece is anodically polarized and immersed. Through a combination of electrochemical reactions, a plasma layer surrounds the workpiece and removes surface peaks [17]. Zeidler et al. (2016) [17] showed a polishing effect of dental Co-Cr crowns and a partial denture framework achieved by plasma electrolytic polishing using macrophotography. Comparative studies using various microscopic methods with respect to the analysis of the surface roughness using atomic force microscopy are largely lacking to date. The aim of the present pilot study was to compare sandblasted, plasma electrolytic and conventional mechanically polished Co-Cr-Mo alloys from the micro- to nanoscale. The originality of this study lies in the fact that we systematically compared polishing methods using stereozoom microscopy, atomic force microscopy (AFM), scanning electron microscopy (SEM) and energy-dispersive X-ray spectroscopy (EDX).

## 2. Materials and Methods

### 2.1. Metal Alloy Specimens

Specimens of two commercially available non-noble cobalt-chromium-based dental alloys were selected for the comparison (see Table 1).

Twenty specimens were polished by conventional mechanical polishing (n = 10 Heraenium^®^ Sun, n = 10 Wironit^®^), and another twenty specimens (n = 10 Heraenium^®^ Sun, n = 10 Wironit^®^) were prepared with plasma electrolytic polishing. Therefore, the excess material left from the metal casting of dental crowns or partial denture frameworks was used as the substrate for comparing the conventional and plasma electrolytic polishing.

For the Wironit^®^ specimen production, a casting wax (Berg Dental, Engen, Germany) for modulation and a precision dental casting investment (Micro, Siladent, Goslar, Germany) for the dental model, prepared with a mixing liquid (Expansion Liquid Type 100, Siladent, Goslar, Germany), were used. For the Heraenium^®^ Sun specimen, another dental casting investment (Cehacast Speed, Hafner, Wimsheim, Germany) was prepared with a specific mixing liquid (Cehacast FS, Hafner, Wimsheim, Germany). The dental model was preheated to 950 °C–1050 °C and cast with the respective metal alloy at approximately 1500 °C in the same dental furnace (MIHM-VOGT, Stutensee-Blankenloch, Germany). After cooling, the dental models were extracted with a hammer, sandblasted (KaVo Dental GmbH, Biberach/Riss, Germany) and alu-blasted (grain size of 250 µm, HS-Edelkorund, Henry Schein, Langen, Germany). The Heraenium^®^ Sun specimens were also bead-blasted (glass bead size 50 µm, Henry Schein, Langen, Germany). Afterward, the specimens were steam-cleaned at up to 6 bar at a high temperature of up to 164° with a steam cleaner Wasi-Steam 2 (Wassermann Dental-Maschinen, Hamburg, Germany). The specimens were split into halves with a dental laboratory handpiece (Kavo K9, Biberach, Germany) with a cutting disc (HS-Trennscheibe für Metalle, Henry Schein, Langen, Germany).

**Figure 1:**
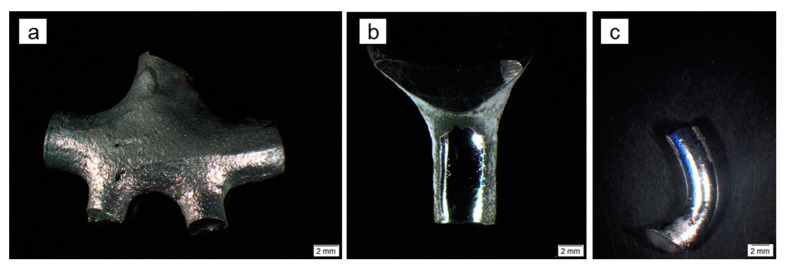
Stereozoom microscopy of the Co-Cr samples, (**a**) a sandblasted Heraenium^®^ Sun specimen, (**b**) a mechanically polished Heraenium^®^ Sun specimen and (**c**) a Wironit^®^ specimen prepared with plasma electrolytic polishing.

Figure 1 illustrates the macroscopic morphology of the samples.

### 2.2. Conventional Mechanical Polishing

First, universal polishers (Ref. 652900303533220, Hager & Meisinger, Neuss, Germany; LOT 2202, Voss Dental, Salzhausen, Germany) and an Al_2_O_3_ corundum abrasive (Ref. 625104107523065, Hager & Meisinger, Neuss, Germany) for the aforementioned dental laboratory handpiece were used for the initial polishing. Afterward, the specimens were rubber-polished (in order) with an abrasive polisher for Co-Cr-Mo alloys (ChromoPol UM, Edenta, Au, Switzerland), rubber polishing wheel (Ref. 43370, Bego, Bremen, Germany), universal polisher (Ref. 652900303533220, Hager & Meisinger, Neuss, Germany) and a rubber polishing tip (Ref. 43370, Bego, Bremen, Germany). The high-luster finish was obtained using a mechanical polishing unit (Typ WP-EX 2000, Wassermann Dental Maschinen, Hamburg, Germany), which first used a high-luster polishing brush (Polirapid Germany, Singen, Germany) and a gold polishing paste (Hera GPP 99, Kulzer, Hanau, Germany). After that, another high-luster polishing brush and a universal polishing paste (Ivoclar Vivadent, Eilwangen, Germany) were used. The speed varied from 5000 to 35,000 rpm, according to the manufacturer’s recommendation. The directions of the polishing constantly varied. After polishing, the specimens were put in an ultrasonic cleaning device (Stammopur RD5, Dr. H. Stamm GmbH, Berlin, Germany) and steam-cleaned with a Wasi-Steam 2.

### 2.3. Plasma Electrolytic Polishing

The specimens were treated using an in-house developed plasma electrolytic polishing system. The polishing system was operated with a bath of ammonium sulfate solution (5%*_w/v_*, el. conductivity of 110 mS/cm), applied voltage of 320 V and direct current at 70 °C. Each specimen was placed with a gripper, acting as an anode. The stainless steel tub was the cathode. The control software for data recording was LabView-based (National Instruments Germany, Munich, Germany). The specimens were treated for 5 min. The electric current acts as a heat source such that the electrolyte bath develops a steam layer near the specimen surface which drives the etching process (anode attracts hydrogen ions). After treatment, the deposited salts at the specimen surface were cleaned with a cotton cloth. In addition, a treatment time series (5, 10, 20, 30 min) was recorded in an exemplary manner for 4 Heraenium^®^ Sun and Wironit^®^ specimens.

### 2.4. Stereo, Scanning Electron (SEM) and Atomic Force (AFM) Microscopy

The surface structure of the specimens, before and after polishing, was documented with a Stereozoom SZX 10 with KL1500LCD (Olympus, Hamburg, Germany). For SEM, high-resolution images (20× to 5000× magnification) of the specimens were obtained with a Quanta FEG 250 SEM (FEI Company, Eindhoven, The Netherlands). It was operated in high vacuum mode (HighVac-SEM), 10 kV–20 kV acceleration voltage and machine-specific spot size Spot 3. Each specimen was placed on an SEM plate with a carbon adhesive disc, Leit-Tab, and copper adhesive tape (Piano, Wetzlar, Germany). Dust particles were cleared off the specimen surface with pressurized air (Druckluft 67; CRC Industries Europe BVBA, Zele, Belgium). For AFM, the specimen surface morphology and roughness were ascertained with a NanoWizard 1 AFM, NanoWizard Control Software v.5.0.97, and Data Processing v.5.0.97 (JPK instruments, Berlin, Germany). The AFM cantilever was an Ultrasharp CSC37/no Al (MikroMasch, Tallinn, Estonia) with a total tip height of 12 µm. Samples were obtained in contact mode. Operating parameters were a cutoff frequency of 150 Hz, a gain factor of 0.05, an operating point of 0.3 V at V_sum_ = 1.3 V–1.5 V, a scan size of 100 × 100 µm^2^ and 30 × 30 µm^2^, a line speed of 0.3 Hz and a resolution of 512 × 512. The AFM software has a corresponding highpass filter reliant on the scanline length. Thus, complex form and waviness do not affect the calculation of the surface roughness values. All surface roughness values were calculated from 10 cross-sectional height profiles within the 100 × 100 µm^2^ scan fields.

### 2.5. Energy-Dispersive X-ray Spectroscopy (EDX)

For the determination of surface homogeneity of the polished samples, an EDAX TEAM detector system (EDAX/AMETEK, Berwyn, PA, USA) was used in conjunction with a Quanta FEG 250 (FEI Company, Eindhoven, The Netherlands). EDX was performed along a predefined line with a line scan of 1 µm width and 400 measurement points per line scan averaged over 8 scans. The accelerating voltage was 20 kV. The element selection was restricted to Mo, Cr, Co, Fe and W.

### 2.6. Statistical Analysis

Statistical analysis was performed using the IBM SPSS Statistics Software Version 29.0.1.0 (IBM, Chicago, IL, USA). All mean surface roughness values Ra (average of 10 line measurements) were checked for normality with a Shapiro–Wilk test. Pairwise comparisons of Ra values for different methods were analyzed with an independent-sample Kruskal–Wallis test. The significance level is 0.05.

## 3. Results

### 3.1. Sandblasted and Mechanically and Plasma-Polished Dental Co-Cr Alloys: SEM

**Figure 2:**
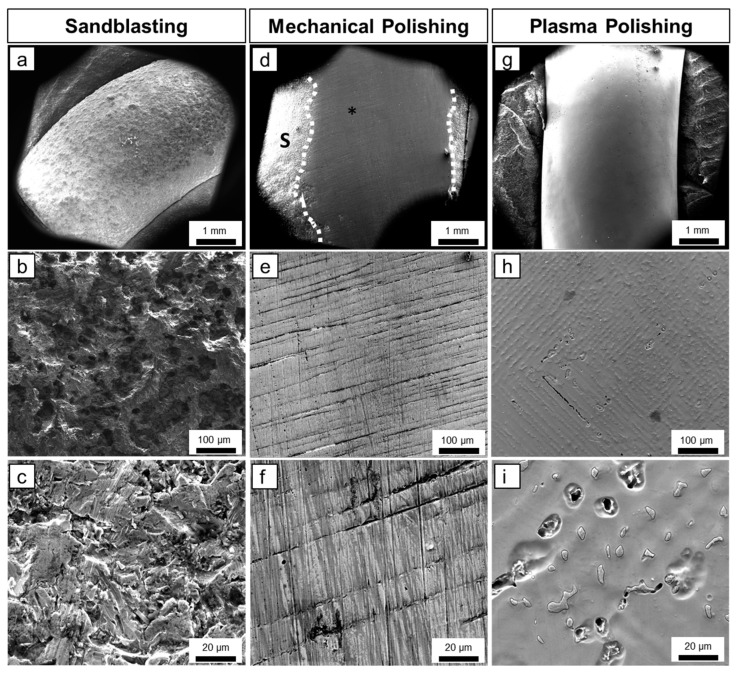
SEM micrographs revealing the comparison of sandblasted, mechanically polished and plasma electrolytically polished Co-Cr alloys. (**a**) Overview of sandblasted Wironit^®^ depicting a rough surface morphology. (**b**) Close-up view of sandblasted Wironit^®^ showing a rough and fissured surface. (**c**) High magnification of sandblasted Heraenium^®^ Sun reveals an irregular surface with ridges, edges and deeper porosities. (**d**) Overview of mechanically polished Heraenium^®^ Sun depicting a clear border between mechanically polished (*) and sandblasted area (S). (**e**,**f**) High magnification of mechanically polished Heraenium^®^ Sun reveals a smooth surface with numerous polishing marks. (**g**) Overview of plasma electrolytically polished Wironit^®^ showing a comprehensive smooth surface. (**h**) Close-up of plasma electrolytically polished Wironit^®^ showing a smooth surface with numerous inhomogeneities. (**i**) High magnification of plasma electrolytically polished Wironit^®^ reveals a smooth surface with interspersed insular structures.

The electron micrographs of Co-Cr- alloys prepared by sandblasting, conventional mechanical polishing and plasma electrolytic polishing are compared in Figure 2. When observed in the SEM, the Heraenium^®^ Sun, as well as the Wironit^®^ specimens, showed similar surface characteristics. As shown in Figure 2a–c, the sandblasted specimen had a rough surface morphology with irregular edges, ridges and deeper porosities. After conventional mechanical polishing (Figure 2d–f), the surface was smoother compared to sandblasting. Numerous polishing marks with changing orientation are obvious. Plasma electrolytically polished surfaces showed a comprehensive smooth surface (Figure 2g) without polishing marks. Higher magnification revealed numerous inhomogeneities and interspersed insular structures.

### 3.2. Sandblasted and Mechanically and Plasma-Polished Dental Co-Cr Alloys: AFM

**Figure 3:**
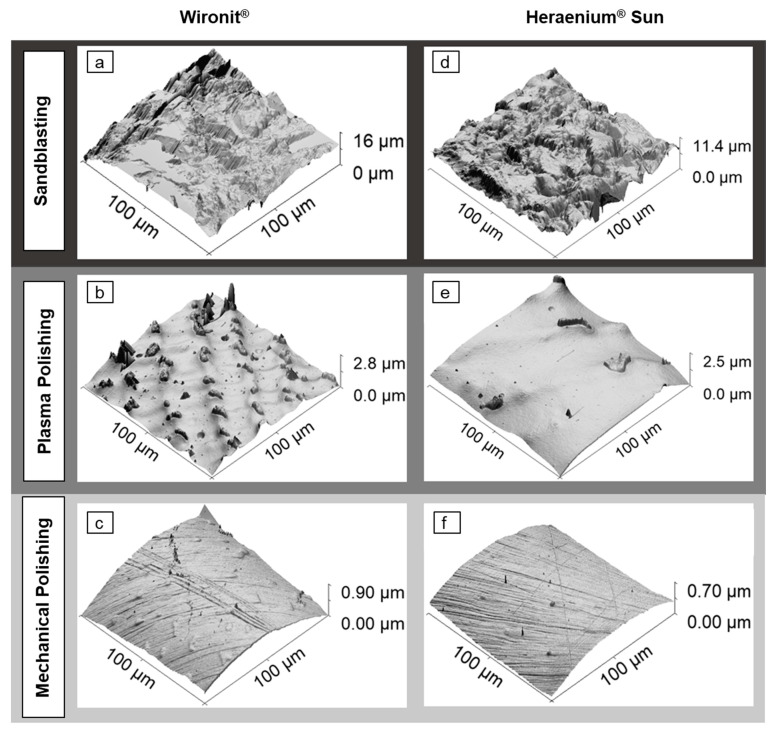
Comparison of 3D AFM images of sandblasted, mechanically polished and plasma electrolytically polished Co-Cr alloys. AFM images are 100 µm in width and height. They are shown with OpenGL lighting for better contrast. (**a**) Sandblasted Wironit^®^ specimen showing a rough surface with irregular porosity. (**b**) Plasma-polished Wironit^®^ specimen showing inhomogeneities and interspersed insular structures. (**c**) Mechanically polished Wironit^®^ specimen showing a smooth surface with numerous polishing marks. (**d**) Sandblasted Heraenium^®^ Sun specimen depicting a rough surface. (**e**) Plasma-polished Heraenium^®^ Sun specimen showing a smooth surface with a few inhomogeneities and interspersed insular structures. (**f**) Mechanically polished Heraenium^®^ Sun specimen showing a smooth surface with numerous polishing scratches.

The 3D AFM images of Co-Cr- alloys prepared by sandblasting, conventional mechanical polishing and plasma electrolytic polishing are compared in Figure 3. The AFM images yield comparable results to the SEM images but also give insights into the heights present in the differently polished samples. One can notice that the overall behavior of Heraenium^®^ Sun and Wironit^®^ is very similar for the different polishing methods. The sandblasted surfaces are rough in appearance (Figure 3a,d). The specimens with plasma electrolytic polishing are interspersed with inhomogeneities in an overall smooth surface (Figure 3b,e). The specimens prepared by mechanical polishing exhibit scratches on a smooth surface that contains small inhomogeneities that are less imposing than the ones in the plasma electrolytic polishing (Figure 3c,f).

**Figure 4:**
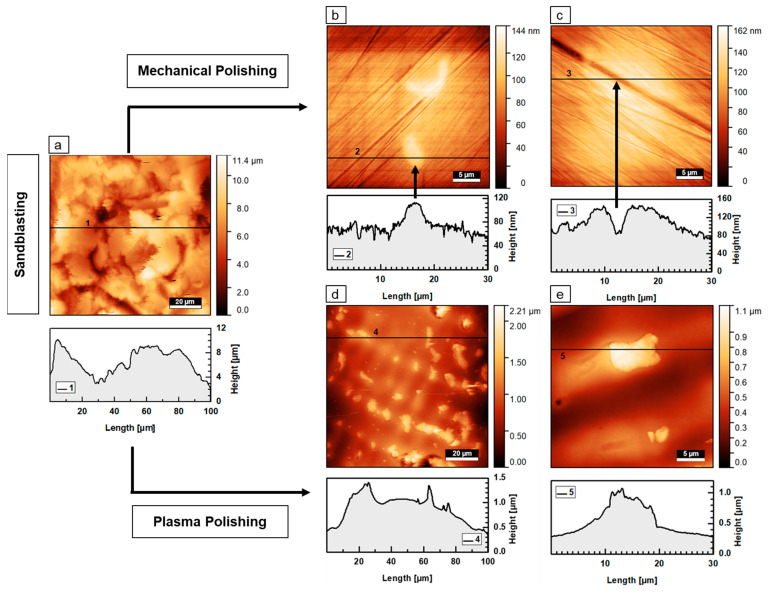
AFM images of Co-Cr specimens and the corresponding horizontal height profile after sandblasting, mechanical polishing and plasma electrolytical polishing. (**a**) Sandblasted Heraenium^®^ Sun specimen (100 µm sized square) showing a surface roughness in the micrometer range. (**b**) The image shows a mechanically polished Wironit^®^ specimen (30 µm sized square). (**c**) The image shows a mechanically polished Heraenium^®^ Sun specimen (30 µm sized square). (**d**) Plasma electrolytically polished Wironit^®^ specimen (100 µm sized square). (**e**) Plasma electrolytically polished Wironit^®^ specimen (30 µm sized square).

Figure 4 presents typical examples of AFM images including their corresponding horizontal height profiles of sandblasted, mechanically and plasma electrolytically polished dental alloys.

The sandblasted specimens were of high surface roughness which is typically in the micron range for both alloys. An example of a sandblasted Heraenium^®^ Sun specimen is presented in Figure 4a, where the rough, heterogeneous surface morphology is visible. A horizontal height line profile is indicated by (1), which shows a variation in height above seven microns. Looking at the microstructure of a Wironit^®^ specimen processed with conventional mechanical polishing, we see in Figure 4b that a smooth surface with regular, patterned islands and numerous polishing marks is suggestive of this polishing method. An example of such an island is given by the black arrow in Figure 4b. Here, the island has a length of about 5 µm, a width of about 2.5 µm and a maximum height of about 30 nm. The scratches seen with SEM are also apparent and are the lowest structures. The overall height of the sample’s surface is 144 nm (Figure 4b), about 100 times lower than the heights seen in the sandblasted specimen with about 10 µm. An example of a deep polishing mark is depicted in Figure 4c. The surfaces obtained with plasma electrolytic polishing appeared for both alloys smooth without scratches, as shown for a Wironit^®^ specimen in Figure 4d. However, numerous interspersed periodic islands are found. An island with a horizontal height profile is presented in Figure 4e. Here, the island had a height of about 0.5 µm, a length of about 7.5 µm and a width of about 5 µm.

**Figure 5:**
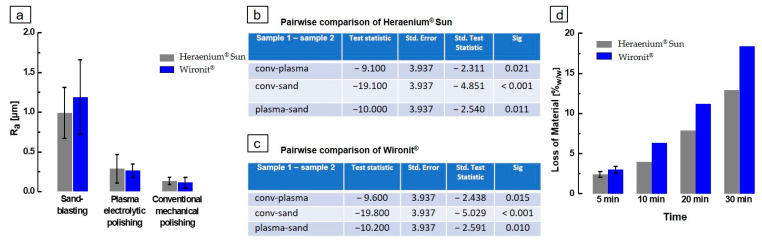
Statistical analysis of sandblasted, conventional mechanically polished and plasma electrolytically polished Heraenium^®^ Sun and Wironit^®^. (**a**) Comparison of the surface roughness values R_a_. The R_a_ values are in SI (Tables S1 and S2). (**b**) Pairwise comparison of surface roughness values of Heraenium^®^ Sun specimen. The significance level is 0.05. (**c**) Pairwise comparison of surface roughness values of Wironit^®^ specimen. The significance level is 0.05. (**d**) The comparison of loss of material during plasma electrolytic polishing at respective times. Data can be found in SI (Table S3).

The overall comparison of the arithmetic mean roughness R_a_ for specimens treated with sandblasting, conventional mechanical polishing or plasma electrolytic polishing is presented in Figure 5a. All R_a_ values can be found in SI in Tables S1 and S2. The sandblasted specimen had the highest observed roughness (Heraenium^®^ Sun 991 ± 288 nm; Wironit^®^ 1187 ± 331 nm), followed by plasma electrolytic polishing (Heraenium^®^ Sun 288 ± 94 nm; Wironit^®^ 261± 49 nm). The lowest roughness values for R_a_ were found for conventional mechanical polishing (Heraenium^®^ Sun 134 ± 23 nm; Wironit^®^ 114 ± 11). Heraenium^®^ Sun plasma-treated, Wironit^®^ sandblasted and Wironit^®^ plasma-treated mean Ra values were found to not follow a normal distribution (*p* < 0.05). Statistical analysis showed that the surface roughness of mechanically polished specimens (Heraenium^®^ Sun *p* < 0.001, Wironit^®^ *p* < 0.001) and plasma-polished specimens (Heraenium^®^ Sun *p* < 0.011, Wironit^®^ *p* < 0.010) are significantly lower than sandblasted specimens. Conventional mechanical polishing (Heraenium^®^ Sun *p* < 0.021; Wironit^®^ *p* < 0.015) provided significantly lower surface roughness values compared to plasma electrolytic polishing (Figure 5b,c). The treatment time in the plasma electrolytic bath was limited to 5 min because, with more time spent in the bath, more alloy was lost, as shown in Figure 5d. The amount of alloy lost is comparable at 5 min for both Heraenium^®^ Sun and Wironit^®^ but becomes increasingly divergent with higher losses of Wironit^®^ to the bath.

### 3.3. Sandblasted and Mechanically and Plasma-Polished Dental Co-Cr Alloys: EDX

**Figure 6:**
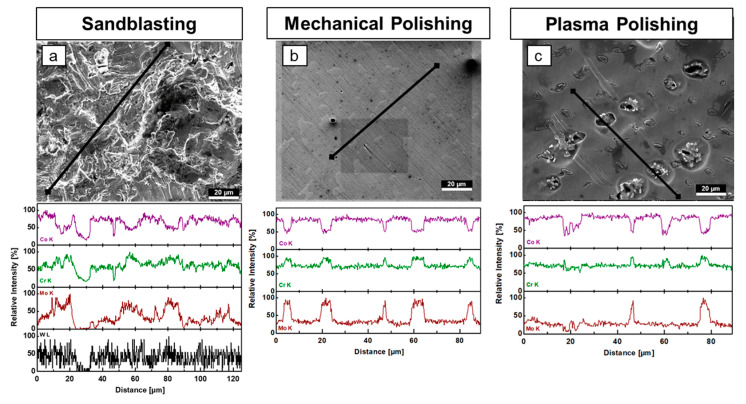
Comparison of EDX measurements (**a**) Sandblasted Wironit^®^ specimen analyzed with EDX along a defined line. Mo, Cr, Co and W were selected and are shown in corresponding concentration curves (1000-fold magnification; SEM and EDX). (**b**) A conventional laboratory-polished Wironit^®^ specimen was analyzed with EDX along a defined line. Co, Cr and Mo were selected and shown in corresponding normalized concentration curves (1000-fold magnification; SEM and EDX). (**c**) Plasma electrolytically polished Wironit^®^ specimen analyzed with EDX along a defined line. Co, Cr, Mo and W were selected and are found in corresponding normalized concentration curves. SEM image is shown at 1000-fold magnification.

Upon further analysis of a Wironit^®^ sample with EDX, a heterogeneous surface distribution of Cr, Co, Mo and W is found, shown in Figure 6a. Here, a line scan, from lower left to upper right, yields the relative intensities of Cr, Co, Mo and W (for absolute intensity, see Supporting Information, Figure S1) that shows a similar behavior for Cr and Co and a slightly deviating one for Mo. W is only found in small traces, as expected from the composition of PDA (Table 1). Along the line scan, one notices an association at a distance of 20 µm, 60 µm and 80 µm of Cr and Mo with relatively high intensities with concurrently decreased intensity in Co, corresponding to bright ridges in the REM image. The minimum EDX relative intensity for Co, Cr, Mo and W at 30 µm is represented in the REM image as a darker valley. Here, between 25 µm and 30 µm exclusively, a signal in Co and Cr is seen. A distance of about 35 µm to the end of the line scan shows an approximately constant association of Co and Cr in the relative intensity of about 75% with a fluctuation width of about 25%. The relative intensity of Mo in the same range varies immensely, indicating a rather heterogeneous spread of elements.

Upon EDX analysis of a conventional mechanically polished Wironit^®^ specimen, the results in Figure 6b show a line scan (black line) from the upper right to the lower left of the SEM image. Here, the relative intensities in Cr and Mo show coinciding intensity peaks, while Co exhibits at Cr and Mo peaks a decrease in concentration indicating a local change in the alloy composition. A comparison with the SEM image shows that the island area is associated with the Cr and Mo peaks and the Co decrease. The EDX analysis of a Wironit^®^ specimen prepared by plasma electrolytic polishing presented in Figure 6c shows a behavior previously seen in Figure 6b. In the diagonal line scan (black line), from bottom to top, the relative intensity peaks in Cr and Mo coincide with troughs in Co within the embedded island. An exposed island at a distance of 60 µm has a similar EDX intensity peak in Co and Cr, but the Mo peak contribution is lost by exposure.

## 4. Discussion

This study is novel regarding the systematic and detailed comparison of dental Co-Cr alloys prepared by sandblasting, conventional mechanical polishing and plasma electrolytical polishing. A combination of various microscopic examination methods (including stereozoom microscopy, AFM and SEM), surface roughness measurements and EDX analyses were used. Such systematic comparative studies are largely lacking to date, particularly related to plasma electrolytically polished dental Co-Cr alloys. To our knowledge, this is the first study to compare conventional mechanical polishing and plasma electrolytic polishing of dental Co-Co alloys by using various examination methods mentioned above.

The surface of dental alloys must be as smooth as possible to limit biofilm formation [9]. As reported by Bollen et al. (1997) [10], some in vivo studies suggest a threshold surface roughness R_a_ of 0.2 µm. A surface roughness below 0.2 µm induces no more reduction in biofilm formation. Our study has shown that the surface roughness of the sandblasted specimens is in the micron range for both Heraenium^®^ Sun and Wironit^®^. They had the highest observed roughness (Heraenium^®^ Sun 991 ± 288 nm; Wironit^®^ 1187 ± 331 nm). Our observation that sandblasted dental alloys show a rough surface with ridges, edges and deeper porosities is in agreement with the literature [13].

In the literature, microscopic observation revealed a typical dendritic/interdendritic microstructure of dental Co-Cr alloys [14]. After conventional mechanical polishing and plasma electrolytical polishing, it was possible in our study to see the alloy’s microstructure on the polished surfaces. Our results are consistent with other studies which have shown that the Co-Cr alloys consist of smooth dendritic regions and interdendritic regions containing clusters of nanocrystallites forming outstanding islands [18]. The islands’ surface size ranged from 4 to 80 µm^2^, and the islands’ height ranged from 90 to 160 nm [14]. Our study is in agreement because we can confirm the described sizes of the islands.

Conventional mechanically polished specimens have a smooth surface with numerous polishing marks [14]. This is in good agreement with our results. A reduction in surface roughness is found for both plasma electrolytic polishing (Heraenium^®^ Sun 288 ± 94 nm; Wironit^®^ 261 ± 49 nm) and conventional mechanical polishing (Heraenium^®^ Sun 134 ± 23 nm; Wironit^®^ 114 ± 11). Studies using AFM with respect to the surface roughness analysis of plasma-polished dental alloys are largely lacking to date. Only one study, to our knowledge, has investigated mechanically polished Co-Cr dental alloys using AFM [12]. Unfortunately, the surface roughness values are not comparable because, in the literature, the surface roughness values (S_a_) were measured from rectangular areas. In contrast, our study used surface roughness values (R_a_) from line scans.

Plasma polishing is not as effective at clearing the surface inhomogeneities, likely due to the different alloy compositions. Plasma electrolytic polishing reduces the R_a_, treatment time and required equipment of the examined dental alloys, Wironit^®^ and Heraenium^®^ Sun. Residues from polishing pastes and scratches from the polishing, common to conventional mechanical polishing, are not found due to the chemical nature of the process, yielding a clean and smooth surface. Plasma electrolytic polishing is less effective in reducing Ra than conventional mechanical polishing. The obtained R_a_ with conventional mechanical polishing is sufficient to contain biofilm formation. The obtained R_a_ with plasma electrolytical polishing is slightly above the published threshold surface roughness of about 0.2 µm for biofilm formation [10,19]. It stands to reason that with improvements in the pre-processing of the alloy samples, a comparable surface quality, below the surface roughness threshold for biofilm formation of 0.2 µm, is within reach of this method. Additional microbiological and in vivo experiments would be favorable to explicitly demonstrate biofilm formation inhibition.

Some reports in the literature [18,20,21] are of the opinion that dendritic and interdendritic areas differed in structure but only slightly in composition. Dendritic regions are described as cubic close-packed crystal lattice structures, while interdendritic regions are described as hexagonal-packed lattice structures. In contrast to the literature, we show a major difference in the composition between the islands and the smoother surroundings. This was illustrated with the EDX analysis (Figure 6). In the EDX profiles of the polished specimen, the relative intensity of Co decreased on an island, while the relative intensity of Cr and Mo increased. Because this tendency is similar for both polishing methods (mechanical, plasma), it is very unlikely an artifact is induced by the surface morphology. Even in the EDX profile of the sandblasted specimen, Co, Cr and Mo fluctuations in the element composition show a similar tendency.

The main limitation of the experimental result is the number of specimens (surface roughness n = 10; EDX n = 1). In addition, we did not explore the polishing load during conventional polishing. The main clinical implication of our experimental result is that conventional mechanical polishing provided lower surface roughness values compared to plasma electrolytic polishing and is considered the gold standard.

## 5. Conclusions

In this work, we compared the surface roughness (R_a_) of the dental alloys, Heraenium^®^ Sun and Wironit^®^, obtained from conventional mechanical and plasma electrolytic polishing, and we compared it to an initial sandblasting. The sandblasted samples were the roughest (Heraenium^®^ Sun 991 ± 288 nm; Wironit^®^ 1187 ± 331 nm), followed by plasma electrolytic polishing (Heraenium^®^ Sun 288 ± 94 nm; Wironit^®^ 261 ± 49 nm). Lower values are found for conventional mechanical polishing (Heraenium^®^ Sun 134 ± 23 nm; Wironit^®^ 114 ± 11 nm). The time needed for the plasma electrolytic polishing is short with only 5 min compared to the conventional method, where extensive manual polishing was required. The material loss for the plasma electrolytic treatment for Heraenium^®^ Sun and Wironit^®^ was less than 4%*w*/*w* for a 5 min treatment time. Surface investigation with AFM and SEM revealed a microstructure of small interspersed regular structures (islands) that exhibited a composition differing from the surrounding smooth area. The result of plasma electrolytic polishing is inferior to the quality obtained with conventional mechanical polishing concerning surface roughness. The obtained surface roughness with conventional mechanical polishing is sufficient for biofilm containment. The obtained surface roughness with plasma electrolytic polishing is slightly above for biofilm containment. R_a_ depends on the initial structure and roughness of the sample, and adequate process parameters must be established to optimize material loss. Overall, our results suggest that plasma electrolytic polishing is a viable polishing method compared to conventional mechanical polishing with a substantial reduction in treatment time. Further research is necessary to improve the plasma polishing parameters to further decrease the surface roughness values. 

## Figures and Tables

**Table 1:** Non-noble dental alloys chosen by frequent use.

Product Name	Manufacturer	Composition (%*_w/w_*)								
Heraenium^®^ Sun REF:66020652	Heraeus Kulzer, Hanau, Germany	Co	Cr	Mo	Mn	Si	Fe	N	C	W
		43.0	23.5	2.0	0.8	1.0	27.0	0.15	0.1	2.5
Wironit^®^ REF:50030	Bego, Bremen, Germany	Co	Cr	Mo	Si	(Mn and C each <1)				
		64.0	28.6	5.0	1.0					

## Data Availability

The underlying data of this manuscript are available from the corresponding author upon reasonable request.

## Supplementary Materials

The following supporting information can be downloaded at https://www.mdpi.com/article/10.3390/ma16186222/s1, Table S1: Heraenium^®^ Sun sample roughness R_a_ and associated standard deviation ΔR_a_ obtained from AFM analysis (line length of 100 µm) after conventional mechanical polishing, plasma electrolytic polishing, and unpolished sandblasted state; Table S2: Wironit^®^ sample roughness R_a_ and associated standard deviation ΔR_a_ obtained from AFM analysis (line length of 100 µm) after conventional mechanical polishing, plasma electrolytic polishing, and unpolished sandblasted state; Table S3: Sample weight loss due to plasma electrolytic polishing. Figure S1: Sandblasted specimens in the unpolished reference state. (a) An image of an unpolished Heraenium^®^ Sun specimen is shown (1000-fold magnification; SEM). (b) A ravine is found on an unpolished Heraenium^®^ Sun specimen (1000-fold magnification; SEM). (c) A sandblasted Heraenium^®^ Sun (BMA) specimen is analyzed with EDX along a defined line. (d,e) Co, Cr, Mo and W were selected and are shown in corresponding concentration curves (1000-fold magnification; SEM and EDX). (f) Sandblasted Wironit^®^ (PDA) specimen analyzed with EDX along a defined line. Mo, Cr, Co and W were selected and are shown in corresponding concentration curves (1000-fold magnification; EDX). Absolute intensities are shown. Relative intensities are found in Figure 3b,c with the original SEM image.
