# Supplementary material for: Mechanical and Plasma Electrolytic Polishing of Dental Alloys
Source: Materials (Basel). 2023 Sep 15;16(18):6222. doi: 10.3390/ma16186222 (PMC10532733; doi:10.3390/ma16186222)
Supplement: Supplementary file 1 [file materials-16-06222-s001.zip › materials-2585401-supplementary.pdf]

materials

MDPI

Article

Mechanical and Plasma Electrolytic Polishing of Dental Alloys

Katharina Witzke [1,*], Renko Kensbock [1], Caroline Ulrike Willsch [1], Katja Fricke [2], Sander Bekeschus [2,3,†] and Hans-Robert Metelmann [1,†]

1 Department of Oral, Maxillofacial, and Plastic Surgery, Greifswald University Medical Center, Sauerbruchstr., 17475 Greifswald, Germany; renko.kensbock@med.uni-greifswald.de (R.K.); caroline.willsch@aol.de (C.U.W.); metelman@uni-greifswald.de (H.-R.M.)
2 Leibniz Institute for Plasma Science and Technology (INP), Felix Hausdorff-Str. 2, 17489 Greifswald, Germany; fricke@nebula-biocides.de (K.F.); sander.bekeschus@gmail.com (S.B.)
3 Clinic and Policlinic for Dermatology and Venerology, Rostock University Medical Center, Strempelstr. 13, 18057 Rostock, Germany
* Correspondence: katharina.witzke@med.uni-greifswald.de
† These authors contributed equally to this work.

Table S1. Heraenium® Sun sample roughness R_a and associated standard deviation ΔR_a obtained from AFM analysis (line length of 100 µm) after conventional mechanical polishing, plasma electrolytic polishing, and unpolished sandblasted state.

sample	conventional mechanical polishing		plasma electrolytic polishing		sandblasted (unpolished)	
Heraenium®	R_a (nm)	ΔR_a (nm)	R_a (nm)	ΔR_a (nm)	R_a (nm)	ΔR_a (nm)
1	108.41	12.96	170.38	51.28	1144.86	355.40
2	109.07	5.31	207.74	58.14	904.03	236.00
3	105.43	11.75	175.62	39.49	1617.70	628.37
4	76.15	25.24	159.84	60.97	1031.52	327.77
5	158.65	8.33	311.54	100.24	718.83	167.15
6	129.43	13.68	238.01	21.59	607.33	294.26
7	238.00	57.76	196.00	81.90	902.29	233.89
8	154.89	5.49	721.87	288.43	575.20	113.49
9	120.70	18.01	223.22	92.48	1223.10	298.28
10	140.71	70.33	478.80	149.90	1187.77	221.01
**mean**	**134.14**	**22.89**	**288.30**	**94.44**	**991.26**	**287.56**

Table S2. Wironit® sample roughness R_a and associated standard deviation ΔR_a obtained from AFM analysis (line length of 100 µm) after conventional mechanical polishing, plasma electrolytic polishing, and unpolished sandblasted state.

sample	conventional mechanical polishing		plasma electrolytic polishing		sandblasted (unpolished)	
Wironit®	R_a (nm)	ΔR_a (nm)	R_a (nm)	ΔR_a (nm)	R_a (nm)	ΔR_a (nm)
1	186.64	18.08	242.12	65.00	994.05	139.25
2	140.24	18.58	242.47	49.33	2397.50	917.81
3	223.06	9.08	248.45	26.95	836.43	284.73
4	140.16	11.87	233.52	47.91	1316.90	165.60
5	132.40	8.70	186.99	35.03	1161.86	341.81
6	53.02	7.15	492.69	102.29	1036.10	649.67

7	124.06	21.22	245.89	36.25	976.19	223.32
8	40.93	5.96	270.78	61.55	1390.50	228.28
9	35.75	3.22	235.31	42.94	1031.51	235.24
10	67.73	3.33	214.97	20.72	732.10	122.43
**Mean**	**114.40**	**10.72**	**261.32**	**48.80**	**1187.31**	**330.81**

Table S3. Sample weight loss due to plasma electrolytic polishing.

sample	time (min)	weight before plasma polishing (g)	weight after plasma polishing (g)	weight difference (g)	relative difference (%)
BMA 1a	5	2.644	2.586	0.058	2.194
BMA 2a	10	6.600	6.339	0.261	3.955
BMA 3a	20	6.168	5.685	0.483	7.831
BMA 4a	30	3.615	3.148	0.467	12.918
BMA 5a	5	5.844	5.732	0.112	1.917
BMA 6a	5	4.917	4.816	0.101	2.054
BMA 7a	5	2.509	2.443	0.066	2.630
BMA 8a	5	1.786	1.733	0.053	2.968
BMA 9a	5	2.173	2.122	0.051	2.347
BMA 10a	5	2.278	2.221	0.057	2.502
PDA 1a	5	2.492	2.440	0.052	2.087
PDA 2a	10	1.407	1.318	0.089	6.326
PDA 3a	20	1.975	1.754	0.221	11.190
PDA 4a	30	1.913	1.562	0.351	18.348
PDA 5a	5	1.422	1.378	0.044	3.094
PDA 6a	5	1.486	1.438	0.048	3.230
PDA 7a	5	1.583	1.534	0.049	3.095
PDA 8a	5	1.921	1.862	0.059	3.071
PDA 9a	5	1.305	1.263	0.042	3.218
PDA 10a	5	1.298	1.256	0.042	3.236

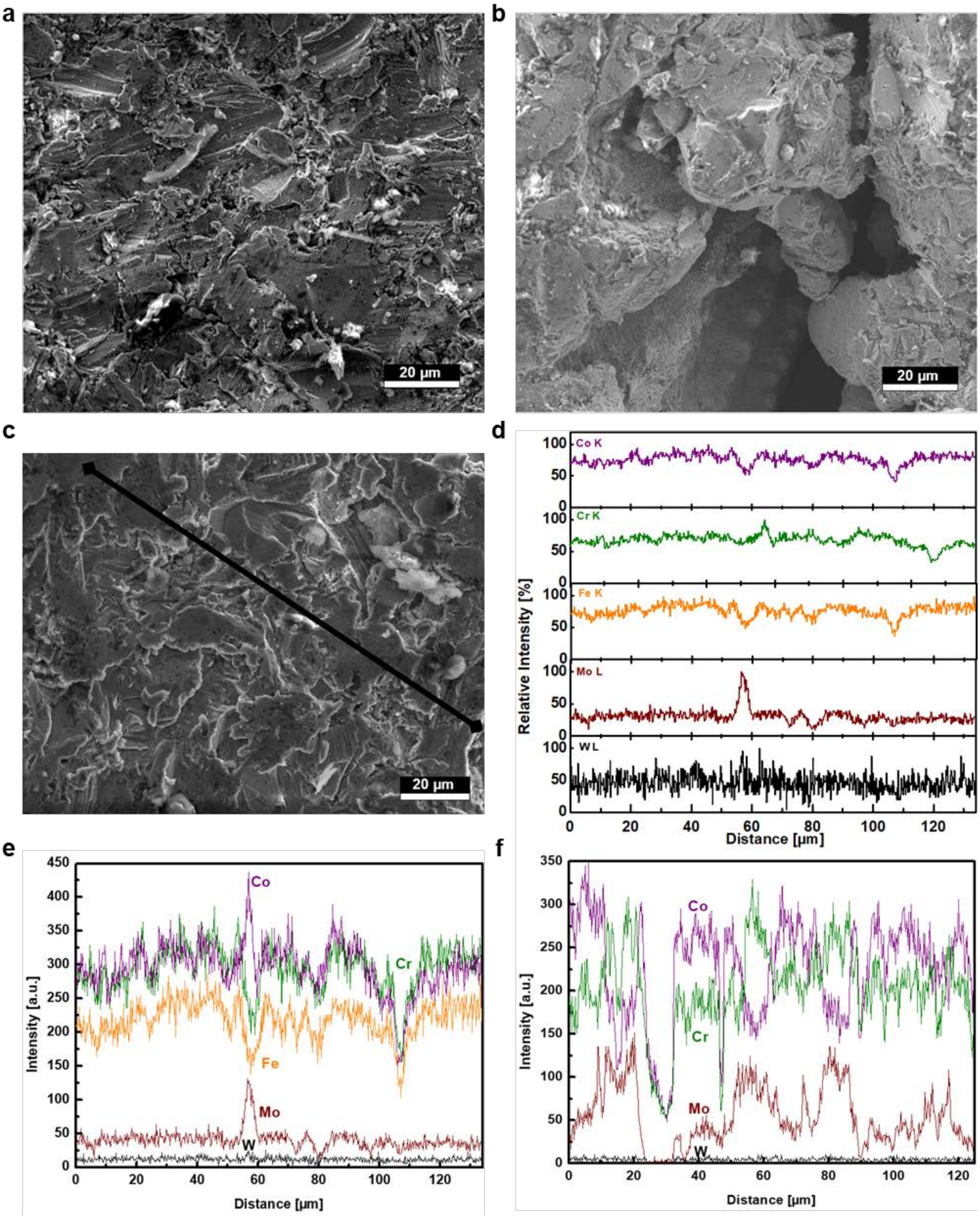

Figure S1. Sandblasted specimens in the unpolished reference state. (a) An image of an unpolished Heraenium® Sun specimen is shown (1000-fold magnification; SEM). (b) A ravine is found on an unpolished Heraenium® Sun specimen (1000-fold magnification; SEM). (c) A sandblasted Heraenium® Sun (BMA) specimen is analyzed with EDX along a defined line. (d–e) Co, Cr, Mo and W were selected and are shown in corresponding concentration curves (1000-fold magnification; SEM and EDX). (f) Sandblasted Wironit® (PDA) specimen analyzed with EDX along a defined line. Mo, Cr, Co and W were selected and are shown in corresponding concentration curves (1000-fold magnification; EDX). Absolute intensities are shown. Relative intensities are found in *Figure 3b -c* with the original SEM image.
